# Clinical and Radiological Findings in Endorectal Migration of a Metallic Ureteral Stent

**DOI:** 10.3390/clinpract16060109

**Published:** 2026-06-11

**Authors:** Szabolcs André, Daniela Dobru, Árpád-Olivér Vida, Miheler Dora, Rares-Florin Vascul, Călin Chibelean, Lorand Tibor Reman, Raul-Dumitru Gherasim, Edva Anna Frunda, Orsolya Katalin Ilona Martha

**Affiliations:** 1 Department of Urology, Mures County Clinical Hospital, P-ța, a Bernády György, Nr. 6, 540072 Târgu Mureș, Romania; szabolcs865@gmail.com (S.A.); calin.chibelean@umfst.ro (C.C.); tibor.reman@umfst.ro (L.T.R.); raul-dumitru.gherasim@umfst.ro (R.-D.G.); orsolya.martha@umfst.ro (O.K.I.M.); 2 Department of Gastroenterology, Mures County Hospital, 540103 Târgu Mureș, Romania; daniela.dobru@umfst.ro; 3 Department of Urology, George Emil Palade University of Medicine, Pharmacy, Science, and Technology of Târgu Mures, 540139 Târgu Mureș, Romania; 4 Department of Obstetrics and Gynecology, Clinical County Emergency Hospital Mures, 540072 Târgu Mureș, Romania; dmiheler@gmail.com; 5 Institution Organizing University Doctoral Studies (IOSUD), George Emil Palade University of Medicine, Pharmacy, Science, and Technology of Târgu Mures, 540139 Târgu Mures, Romania; vascul.rares-florin.25@stud.umfst.ro (R.-F.V.); anna.frunda@gmail.com (E.A.F.)

**Keywords:** cervical cancer, ureteral obstruction, stent migration, ureteral stent complications, rectal fistula

## Abstract

Hydronephrosis caused by malignant ureteral obstruction or radiotherapy-induced ureteral stenosis is a frequent complication in patients with cervical cancer. Effective management requires continuous urinary drainage, which can be achieved either internally through ureteral stent placement or externally via percutaneous nephrostomy. Among available devices, the Allium^TM^ fully covered nitinol mesh ureteral stent is designed to treat ureteral or urethral strictures while allowing safe and easy removal. However, serious complications have been reported, including uretero-enteric, uretero-arterial, and uretero-vaginal fistulas, pseudoaneurysm, ureteral perforation and sepsis. We report the case of a 44-year-old woman diagnosed in 2020 with stage IIIC1 cervical cancer (FIGO classification) who underwent surgery followed by adjuvant radiotherapy. In 2021, a right metallic ureteral stent was placed to treat ureteral obstruction. Two years later, she presented with right lumbar pain, and abdominal ultrasonography revealed grade III right hydronephrosis. CT scan demonstrated migration of the metallic ureteral stent into the rectal wall. Endoscopic extraction of the migrated stent was successfully performed via colonoscopy. Retrograde pyelography and CT imaging confirmed the presence of a recto-ureteral fistula. A 6 Ch/26 cm double-J ureteral stent was subsequently placed with good positioning and drainage. At the six-month follow-up, replacement of the double-J stent was performed. Imaging studies showed only minor residual hydronephrosis. Although metallic ureteral stents are effective for managing malignant ureteral obstruction, particularly in complex oncologic cases, they are not free of severe complications. The risk appears increased in patients who have undergone radiotherapy, emphasizing the need for careful monitoring and long term follow-up.

## 1. Introduction

Cervical cancer remains an important malignancy worldwide and may cause ureteral obstruction through tumor invasion, postoperative fibrosis, or radiotherapy-related ureteral stenosis [1]. It often leads to malignant upper urinary tract obstruction, uretero-hydronephrosis, caused by intrinsic, intramural or extrinsic compression from the malignant disease itself or the fibrosis following radiotherapy or the devitalization, lesion of the ureter during radical hysterectomy [2]. This complication is associated with a poor prognosis, leading to a significant rate of mortality. In these cases the decompression of the renal unit, in order to improve renal function, quality of life and overall survival is of utmost importance. Decompression can be managed internally by the placement of a ureteral stent or externally by means of nephrostomy and should provide uninterrupted urinary drainage [2,3].

These decompression procedures have the following complications: in case of nephrostomy; urinary tract infection, even urosepsis, haematuria, obstruction or dislocation while in case of stenting; stent failure, obstruction, dislocation, calcification, bladder irritation, haematuria, infection, etc.

In uro-oncology Metallic Allium stents, designed to provide long-term patency and high radial force, are frequently used, with the concept of rebuilding a normal epithelium around the stent. One of the most important features of these stents, intended for temporary use, is the capacity to remove them easy and safe [4]. Although Allium stents may be effective for ureteral strictures, reported complications include stent migration, obstruction, and fistula-related complications, particularly in complex cases [3,4]. For patients with advanced cancer and ureteral obstruction, regular stent exchange offers an efficacious way to preserve kidney function and avoid dialysis. Since many of these patients have a short life expectancy, they typically pass away before their renal health fails. Consequently, treatment should emphasize infection control strategies to minimize complications during their remaining time [5].

Compared with conventional polymeric double-J stents, metallic ureteral stents provide greater radial strength and longer functional patency, making them more suitable for malignant ureteral obstruction caused by extrinsic compression. Polymeric stents usually require exchange every 3–6 months and may fail more frequently because of tumor-related or fibrosis-related compression. In contrast, metallic stents can often remain functional for 6–12 months, but they may be associated with specific complications, including migration, difficult retrieval, encrustation, and management challenges in irradiated or anatomically altered pelvic tissues. However, metallic ureteral stents (MUS) are associated with specific risks such as difficult retrieval due to encrustation and potential for migration [6,7]. Therefore, the choice between polymeric and metallic stents should be individualized according to obstruction severity, expected survival, prior radiotherapy, infection risk, and feasibility of follow-up [7,8].

We report the case of a patient with a history of cervical cancer who developed migration of a metallic ureteral stent into the rectum through a uretero-rectal fistula after treatment for right ureteral stenosis. Migration of a metallic Allium ureteral stent into the rectum represents an exceptionally rare and clinically significant complication.

## 2. Case Presentation and Results

A 44-year-old female patient diagnosed with stage III C1 cervical cancer according to FIGO classification 2018 (tumor reaching the lower third of the vagina, extension to the pelvic wall, with pelvic lymph nodes) underwent surgical management (radical hysterectomy with pelvic lymphadenectomy) and adjuvant radiotherapy in 2020 [1].

In 2021, during routine follow-up, sequential CT scans revealed severe right ureteral stenosis. Based on these findings, intravenous urography was performed, confirming the stenosis. (possibly as a complication of radiotherapy). Blood tests were performed to assess renal function and identify possible infection or urosepsis. Serum creatinine was elevated because the stenosis was causing significant hydronephrosis 192 µmol/L (2.17 mg/dL), white blood cell (WBC) count of 14,000 cells/mm^3^ that indicates a significant inflammatory response, typically suggesting a progression to right acute pyelonephritis. A clear indication was established for endourological decompression, and a self-expanding metallic Allium ureteral stent was inserted to achieve decompression of the obstruction. Following a period of monitoring and further conservative management, the patient was discharged home with a normal creatinine level (0.93 mg/dL) and without fever.

**Figure 1:**
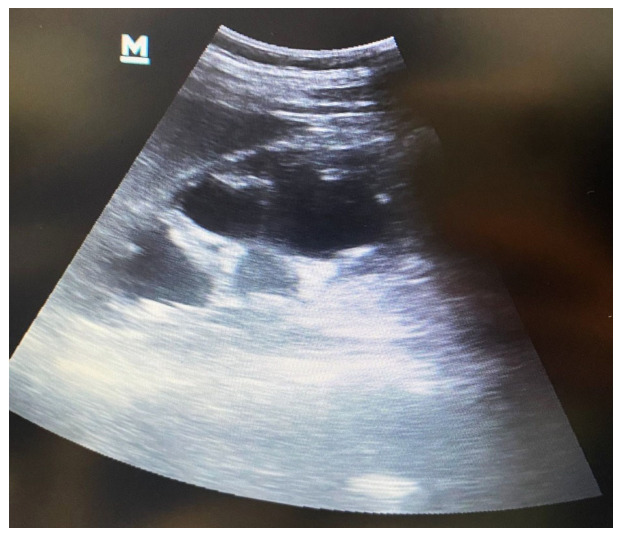
Abdominal ultrasound showed grade 4 right hydronephrosis.

In 2023, the patient was admitted to our emergency department for right-sided lumbar pain, fever of 38.7 °C, pneumaturia. Abdominal ultrasound revealed grade 4 of right hydroureteronephrosis (SFU classification) (Figure 1). Laboratory evaluation demonstrated an elevated serum creatinine level of 201 μmol/L (2.27 mg/dL), consistent with impaired renal function. Urinalysis revealed no hematuria.

**Figure 2:**
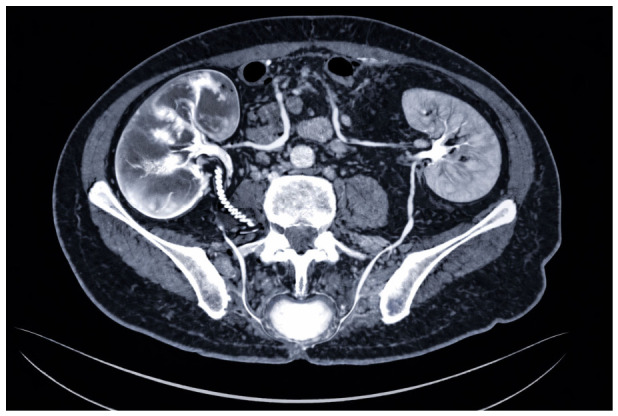
Contrast-enhanced CT scan showing the dislocated, migrated right ureteral stent.

The patient underwent an urgent contrast-enhanced CT scan of the abdomen and pelvis, demonstrating the migration of the right metallic ureteral stent into the rectal wall (Figure 2).

**Figure 3:**
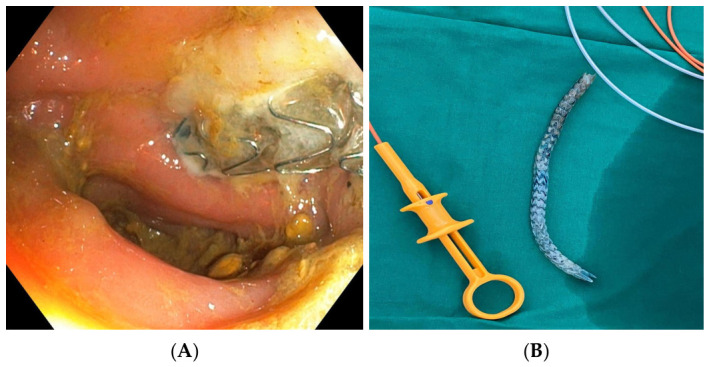
Endoscopic visualization and removal of the migrated metallic ureteral stent. (**A**) The migrated metallic ureteral stent protruding into the rectal wall and covered by necrotic and ulcerated endorectal mucosa. (**B**) Removed metallic ureteral stent.

After the colonoscope was advanced into the rectum, the metallic Allium stent was visualized protruding through the rectal wall and was partially covered by necrotic and ulcerated endorectal mucosa (Figure 3A). The distal end of the stent was grasped with rat-tooth forceps, and the stent was then mobilized by gradual collapse along its longitudinal axis using gentle, continuous traction. During removal, particular care was taken to avoid mucosal injury from the sharp edges of the stent. The ureteral stent was successfully removed endoscopically in one piece (Figure 3B).

After the removal of the migrated stent, ureteropyelography was performed, which visualized the recto-ureteral fistula and the pelvic ureteral stenosis. To ensure urinary drainage, a 6 Ch/26 cm double-J ureteral stent was successfully placed in the right ureter and maintained for six months.

**Figure 4:**
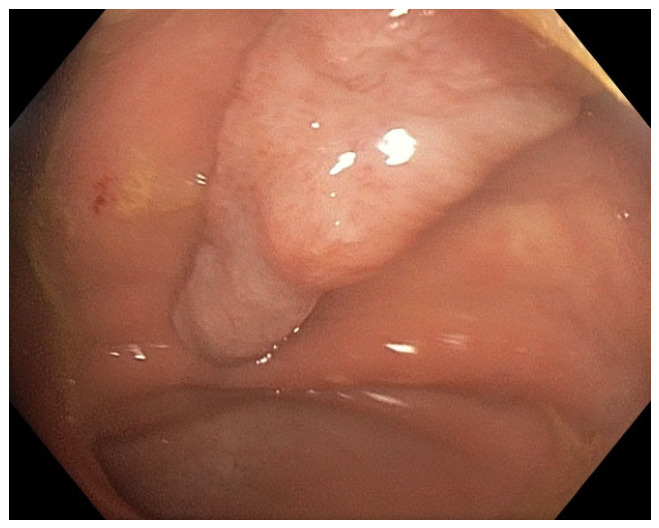
Follow-up colonoscopy performed one month after metallic stent removal, showing healing of the rectal wall and disappearance of the fistula site.

The patient was scheduled for a follow-up colonoscopy after one month, which demonstrated the healed zone of the migrated stent and the resolution of the fistula (Figure 4).

Follow-up and replacement of the double-J ureteral stent were performed after six months. Abdominal ultrasound and CT scan identified minor hydronephrosis and no recto-ureteral fistula.

## 3. Discussion

Ureteral obstruction is a well-recognized complication in patients with advanced or treated cervical cancer, most commonly resulting from tumor infiltration, postoperative fibrosis, or radiation-induced strictures [9]. The study endpoints included complications such as infection and fever. Impairment of renal function, as demonstrated by laboratory or imaging findings, was considered indicative of ureteral obstruction [10]. Self-expanding metallic ureteral stents, such as the Allium stent, have been increasingly used in patients with long-term or refractory ureteral strictures due to their longer patency rates and resistance to external compression compared with conventional polymer stents [10].

Despite these advantages, metallic ureteral stents are not free of complications. Stent migration is relatively uncommon, but may occur, particularly in the presence of altered pelvic anatomy following surgery and radiotherapy, and represents a clinically significant complication. Stent failure was also defined as unanticipated stent exchange or nephrostomy placement for signs of ureteral obstruction [10]. Reported complications included malposition, migration, insufficient relief of obstruction, encrustation, stent fracture, urethral erosion or fistula formation, prolonged stent retention due to neglect or oversight, as well as hematuria and pain [11].

In the presented case, surgery followed by radiotherapy contributed to local tissue weakness and abnormal anatomical relationships between the ureter and adjacent organs, predisposing to stent displacement and erosion. The metallic stent migrated distally and became embedded in the rectal wall without causing perforation, sepsis, or significant bleeding, allowing for a controlled diagnostic and therapeutic approach. Although Allium stents are designed to provide long-term patency and high radial force, this case illustrates that prolonged mechanical pressure, chronic inflammation, local ischemia and major pelvic interventions may lead to gradual erosion of the ureteral wall, allowing transmural migration of the stent into nearby organs, in this case, the rectum [9,12].

Management of ureteral stent migration into the rectum should be individualized based on the patient’s condition and associated complications. Endoscopic removal via colonoscopy is typically the first-line approach, allowing minimally invasive retrieval of the stent using forceps or snares [11].

If retrograde extraction is not feasible, antegrade retrieval through a percutaneous nephrostomy tract may be performed [13]. In more complex cases, such as those involving fistula formation, bowel perforation, or obstruction, laparoscopic or open surgical intervention with stent removal and repair of affected structures may be required [14].

Conservative management may be considered when the stent has already passed, with bladder drainage (e.g., Foley catheter) and bowel rest to promote fistula healing. In severe cases, temporary urinary diversion with nephrostomy tubes may be necessary to allow adequate drainage and facilitate tissue recovery [13,15].

The clinical relevance of rectal migration lies in its potential to cause severe infectious complications, including uretero-enteric fistula formation, sepsis, or peritonitis, while initial symptoms may be nonspecific or subtle. This case emphasizes the importance of long-term surveillance in patients with metallic ureteral stents and a rare but clinically very important complication of the case: the uretero-rectal fistula formation [4,9]. Migration of Allium stents is a rare complication, which can be classified as early and late stent migration. In a previous study including 107 patients treated with Allium ureteral stents, stent migration occurred in 11 patients and required stent removal within eight months of insertion. Stent migration occurred in 10.7% of patients, predominantly among those with mid-ureteral strictures [9]. Another reported case of ureteroarterial fistula treated with an Allium stent showed a more favorable outcome. In that case, intraureteral repair with serial Allium stents successfully controlled acute hemorrhage, maintained hemodynamic stability, and preserved renal function without stent-related complications [16].

This comparison suggests that the clinical context and underlying tissue condition may play a crucial role in determining the safety and effectiveness of Allium stent implantation. In our opinion, fistulization between the urinary tract and the rectum in this case was likely associated with fibrosis and structural deterioration of irradiated pelvic tissues following radiotherapy.

Management of such complications must be individualized. Although surgical removal is often considered, surgery in irradiated fields carries increased morbidity. In our case, colonoscopic removal was successfully performed, avoiding invasive intervention. This case underscores the importance of close follow-up in patients with metallic ureteral stents after pelvic radiotherapy and demonstrates that, in selected cases, endoscopic removal can be a safe and effective alternative to surgery.

## 4. Conclusions

Our case illustrates a rare complication of metallic ureteral stenting, with erosion and migration of the stent into the rectal wall (via uretero-rectal fistula) in a patient with prior pelvic surgery and radiotherapy for cervical cancer. Careful evaluation and a multidisciplinary approach allowed successful colonoscopic removal, avoiding surgical intervention in an irradiated field. Clinicians should remain vigilant for late stent-related complications in high-risk patients, and endoscopic management may represent a safe and minimally invasive treatment option in carefully selected cases.

## Data Availability

The data presented in this study are available on request from the corresponding author due to privacy and ethical restrictions. The data are not publicly available because they contain clinical information related to an individual patient.
